# Multi-Sensor Based State Prediction for Personal Mobility Vehicles

**DOI:** 10.1371/journal.pone.0162593

**Published:** 2016-10-12

**Authors:** Jamilah Abdur-Rahim, Yoichi Morales, Pankaj Gupta, Ichiro Umata, Atsushi Watanabe, Jani Even, Takayuki Suyama, Shin Ishii

**Affiliations:** 1 Department of Dynamic Brain Imaging, Cognitive Mechanisms Laboratories, Advanced Telecommunications Research Institute International, Soraku-gun, Kyoto, Japan; 2 Intelligent Robotics and Communication Laboratories, Advanced Telecommunications Research Institute International, Soraku-gun, Kyoto, Japan; 3 Multisensory Cognition and Computation Laboratory, Universal Communication Research Institute, National Institute of Information and Communications Technology, Soraku-gun, Kyoto, Japan; 4 Department of System Science, Graduate School of Informatics, Kyoto University, Kyoto, Japan; IRCCS Istituto Auxologico Italiano, ITALY

## Abstract

This paper presents a study on multi-modal human emotional state detection while riding a powered wheelchair (PMV; Personal Mobility Vehicle) in an indoor labyrinth-like environment. The study reports findings on the habituation of human stress response during self-driving. In addition, the effects of “loss of controllability”, change in the role of the driver to a passenger, are investigated via an autonomous driving modality. The multi-modal emotional state detector sensing framework consists of four sensing devices: electroencephalograph (EEG), heart inter-beat interval (IBI), galvanic skin response (GSR) and stressor level lever (in the case of autonomous riding). Physiological emotional state measurement characteristics are organized by time-scale, in terms of capturing slower changes (long-term) and quicker changes from moment-to-moment. Experimental results with fifteen participants regarding subjective emotional state reports and commercial software measurements validated the proposed emotional state detector. Short-term GSR and heart signal characterizations captured moment-to-moment emotional state during autonomous riding (Spearman correlation; *ρ* = 0.6, *p* < 0.001). Short-term GSR and EEG characterizations reliably captured moment-to-moment emotional state during self-driving (Classification accuracy; 69.7). Finally, long-term GSR and heart characterizations were confirmed to reliably capture slow changes during autonomous riding and also of emotional state during participant resting state. The purpose of this study and the exploration of various algorithms and sensors in a structured framework is to provide a comprehensive background for multi-modal emotional state prediction experiments and/or applications. Additional discussion regarding the feasibility and utility of the possibilities of these concepts are given.

## Introduction

There is a need for simple (easy to use), robust (not easily damaged), and streamline technologies in order allow individuals to continue personal control of their living environment, without expensive healthcare servicing and/or time-consuming family support. Individuals with disabilities and the elderly often times have difficulties using basic self-propelled wheelchairs, because they lack the motor dexterity and strength to physically propel themselves for long-distances. In addition, wheelchair users face challenges with traveling over rough terrain, inaccessible paths, and other traffic related inconveniences of city-life [1]. In such cases, powered wheelchairs are available to assist with daily-life mobility. However, despite the ease of moving from one-point-to-another, powered wheelchairs require a certain level of motor control. Individuals with significant spinal-cord injury, nervous system diseases, cognitive impairment, and blindness, unfortunately lack the ability to drive themselves using powered PMVs. In addition, individuals whom have the requisite motor control abilities, also find it difficult to use powered wheelchairs because of joystick steering and adaptation to usage of controls. There are other control options for powered wheelchairs besides joystick, such as head or chin control, sip and puff, and other (eye gaze, tongue, head, hand, foot control) [2]. However, these control options can cause unwanted attention in public spaces. Therefore, autonomous wheelchair control has increasingly become an attractive option, despite the expensive cost and immature development of technologies [3, 4].

With the increasing advent of automated vehicle systems, there is a need for understanding and monitoring human emotional states (relaxation, distress) during transport of human passengers. Feelings of discomfort during autonomous wheelchair transport of human passengers, as opposed to self-controlled transport, have not been well studied. However, studies on comfortable movement of autonomous wheelchairs show that subjective comfort can be changed, more specifically increased, by using feedback (psychophysical and questionnaire) from passengers [5–7]. In addition, discomfort during autonomous automobile transport of human passengers has been investigated and shown to be attributed to lack of control and inability to predict movement [8, 9]. Therefore, in order to make PMVs a viable future product geared towards human comfort, like autonomous automobiles, the potential stressors associated with autonomous wheelchair transport need to be investigated and reliable non-invasive monitoring solutions need to be developed.

There are two widely accepted views of stress in relation to psychophysical/cognitive/emotional/behavioral responses; cognitive recognition of stressors and physiological response to stressors. Cognitive recognition of stressors consist of identification of stress via several steps. First, perception of potential stressors are conducted; stressors can arise from external forces via bodily senses (visual, auditory, touch, smell, vestibular) and internal factors via self-internalization (memories, imagined scenarios, etc). Second, primary appraisal is performed to determine whether the stressor is positive, negative, or neutral. If the stressor is negative, an assessment is performed to determine to what extent it is harmful/threatening for the future. Third, a second appraisal is performed to determine what tools/skills one has to cope with overcoming the stressor. Finally, the outcome of coping with the stressor is learned and re-absorbed as a tool/skill for the future [10].

More specifically, the perception of a stressor triggers three phases of response: alarm, resistance, and exhaustion. These three phases are automatic physiological responses to stress. When a stressor is perceived the alarm phase begins, arousal of the sympathetic nervous system starts, heart rate increases, breathing increases, blood pressure increases, muscles become tense, and blood clotting reagents increase. The resistance phase is triggered when the body is unable to maintain sympathetic nervous activity and the parasympathetic nervous system activates. The exhaustion phase begins when stress is prolonged and repeated, symptoms include the initial alarm reaction unabated, and if coping strategies are not utilized serious illness could occur [11]. Both cognitive and physiological explanations often work hand-in-hand, to explain process in which stressors are unconsciously and/or consciously experienced.

Moreover, different emotions are associated with distinct patterns of physiological response. In addition, mimicking emotional states via facial expressions can result in comparable physiological responses similar to actually eliciting the emotion [12]. For example, if a human experiences (or mimics via facial expression) a negative emotion, their body would produce a corresponding physiological response. Where the changes in their physiological/chemical response would induce the same biological symptoms as if their body was experiencing a stressor. Similarly, behavioral feedback literature demonstrates that movement and posture of the body can cause changes in emotional, physiological, chemical response [13]. Emotional, physiological, cognitive, and behavioral states are intrinsically connected and thus the effects of stress influence all of these states [14].

**Fig 1:**
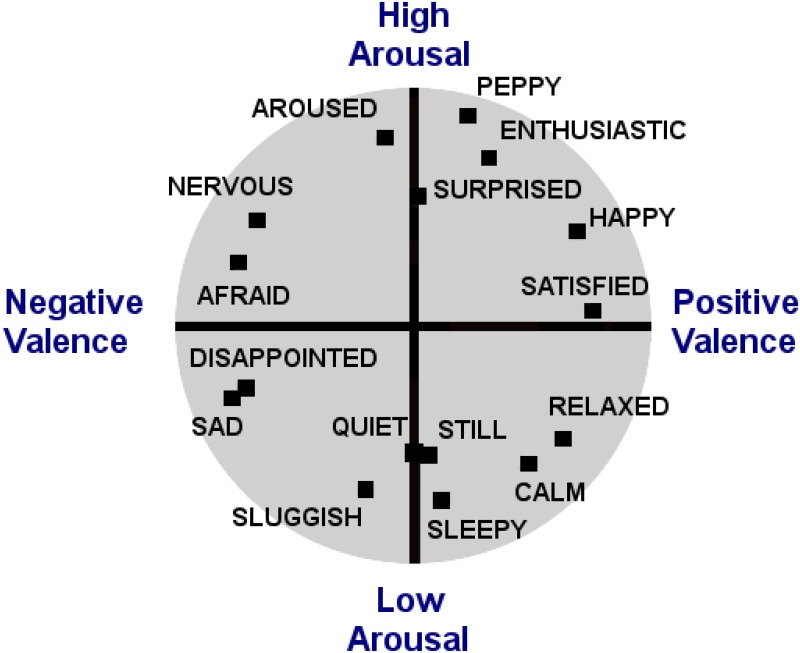
Two dimensional arousal-valence model.

In addition, a perceived stressor can be positive (cause “good” stress, eustress) or negative (cause “bad” stress, distress), and can correspondingly change one’s emotional (arousal-valence) state. Emotional states have been characterized on a two-dimensional plane, arousal-valence circumplex model, where arousal (y-axis) and valence/affect (x-axis) give rise to one’s internal state of being. Fig 1 shows the two-dimensional emotional space in terms of arousal and valence [15, 16]. In the context of this mapping, it is possible to experience neighboring arousal-valence states at the same time, this is referred to as co-occurrences. However, arousal-valence literature supports that, it is not possible to experience two or more non-neighboring emotional states at the same time as one’s internal emotional state is discrete [17].

We define subjective emotional state due to stressors in terms of the accompanied physiological, cognitive, emotional, and behavioral changes due to an environmental/externally perceived stimuli. We only focus on external/environmental stressors, not internal stressors, for relevance to wheelchair operation and simplicity. In other words, a person’s current physiological/cognitive/emotional/behavioral state can be perturbed due to increasing/decreasing demands in arousal and/or valence, in response to perceived external stimuli [11]. The external stimuli/agents/events that are perceived are referred to as stressors [14]. Therefore, the body’s response to external stimuli can be characterized using a stimulus-response paradigm. Under the assumption that the basis of emotional state arises from a stimulus-response conceptualization, stressors can be experienced in four ways under different time-scales: (1) suddenly/time-limited stressor, such as a surprising event; (2) gradually/sequence of stressors, a series of events that occur over an extended period of time; (3) chronic intermittent stressors, events that may occur once a day/week/month; (4) chronic stressors, events that may or may not be caused by a specific event and which continuously persist for a long time [10]. In turn, emotional state perception due to stressors can be quantified in terms of physiological changes and/or by personal report via questionnaires.

Emotional state quantification, due to stressors, via questionnaire has been shown to reliably capture information that the person is consciously aware of at discrete intervals. And, depending on how each question is posed, determines the clarity/reliability of the information. Similarly, emotional state quantification using physiological measurements can reliably capture both unconscious and conscious changes in arousal-valence states, continuously and in real-time [18–20]. However, due to the stimulus-response conceptualization both quantifications are at risk of: habituated/diminished responses due to increased familiarity of the situation [21], inability to identify stressors/disturbances due to lack of a baseline/steady-state of homeostasis, inability to compare stressors for different people.

Physiological measurements are of interest, in contrast to personal report, because PMV systems require continuous and real-time measurements for timely and efficient prediction of emotional state due to stressors. IBI, GSR, body temperature, electromyography (EMG), and EEG are relevant measures for monitoring the body’s response to stressors because these sensors are non-invasive and their interpretations associated with arousal-valence states are straightforward. Standard signal processing methods, such as time and frequency domain analysis, have been used to characterize emotion related measures in order to capture changes in arousal and/or valence in response to stressors [18, 22–24]. However, existing characterizations have not been organized such that inexperienced users of physiological measures (i.e.: engineers, modelers) can wisely select parameters of interest for more advanced modeling usage.

Signal characteristics can be interpreted directly or used to construct a model in order to describe less visible signal trends. Statistical correlations of different signal characteristics and/or task-events are often performed to strengthen direct interpretations and/or confirm task response behavior [18, 25]. Another popular analysis approach is supervised classification, where many relevant signal measures are characterized and used as features to discriminate between two or more internal states [26, 27]. However, the topic of monitoring multiple physiological measurements simultaneously is recent, and the usage and utility (how and when to use) of various signal characterizations in combination remains unclear. Correlation and supervised classification approaches are effective for classical stimulus-driven controlled tasks, where stimulus versus non-stimulus (i.e.: presence of a stressor versus absence of a stressor) periods are defined. However, during complex real-life situations, such as driving, there are no predefined stressor/non-stressor periods. Similarly, each individual may experience stressors differently at different periods of time.

In sum, emotional state monitoring is beneficial for passenger comfort but there are many challenges in terms of signal processing, signal measurement usage, and prediction/modeling of changes in internal state due to stressors. We propose a semi-controlled wheelchair driving experiment, to capture behavior and to address the challenges of emotional state analysis and prediction due to environmental stressors. Previous autonomous wheelchair driving studies only evaluate comfort during shorter and less constrained areas [5–7]. Moreover, previous studies have monitored emotional response to the presence of stressors during realistic events, such as test-taking and virtual reality paradigms, however feedback was not given during rest conditions nor were habituation effects considered [18, 26]. In this work we use physiological sensors to monitor changes in emotional response to the presence of external stressors. To our knowledge, previous internal state monitoring studies in response to stressors have not used physiological measurements as feedback to ensure that participants begin each experimental trial at a similar baseline.

### Physiological correlates of stress

**Fig 2:**
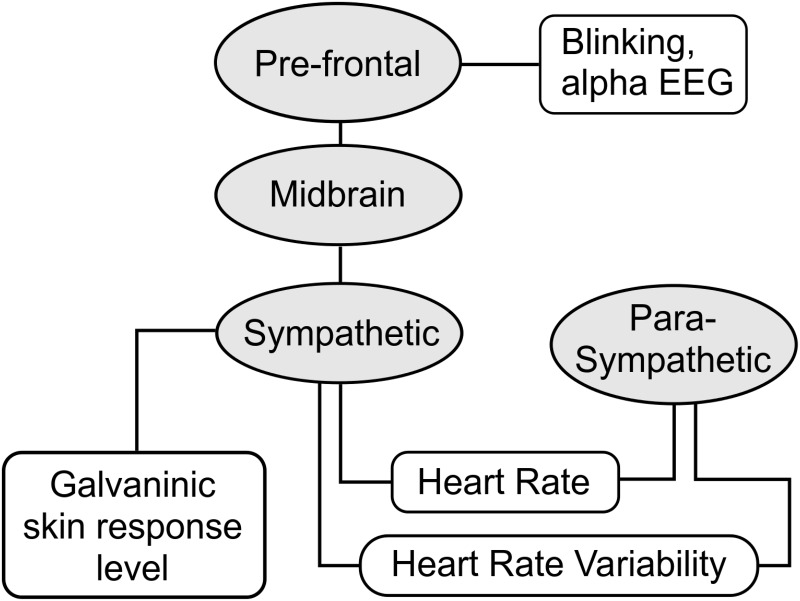
Relationship between stress related neurophysiological structures.

The Autonomous Nervous System is a relevant neurophysiological system that characterizes stress. Fig 2 shows a flow diagram describing the relationship between neurophysiological structures of interest, along with sensor measures of interest. Stress causes the sympathetic system to activate, resulting in an increase of heart rate (HR) (decrease of IBI) and GSR. Heart rate variability decreases as the parasympathetic system is suppressed [18]. In addition, HR activity is associated with respiratory sinus arrhythmia (RSA), which describes variations in HR that occur during breathing. Vagal activity, nerve activity from the brainstem related with homeostasis regulation, links RSA breathing to HR where RSA inhalation suppresses vagal activity causing HR to increase [22]. Similarly, electrodermal activity reflects sympathetic arousal and is often used as an indicator of attention, cognitive effort, and arousal [28]. Reports have also identified EEG alpha frequency band as an indicator of relaxation, and differences in right and left hemisphere activity is linked to stress [29]. Withdrawal/avoidance or approach strategies for coping with stress depending on the direction of asymmetry. Greater left alpha power, in comparison to right alpha power, is associated with withdrawal. And, greater right alpha power, in comparison to left alpha power, is associated with approach behavior [18, 30].

Investigating the correlation between physiological measures has been researched in various combinations: GSR and brain activity [28], asymmetric brain activity and heart rate variability [18], accumulation of GSR and blinking [27], GSR and heart rate variability [31]. In these reports the objective is to determine the origin of another measure’s activity (causality, connectivity), or to find similar patterns of time and/or frequency change between measures. In addition, many of these reports focus on general characterizations of physiological measures under controlled stationary situations.

Existing physiological measurement characterizations can be organized in terms of describing changes in emotional state over short/quick and long/slower time-scales.

Long-term related parameter characterizations are often composed using the mean or grouping methods and frequency domain analysis, in order to capture slower changes. Slow timescale information is useful for describing non-reactive emotional/behavioral responses to stressors. Real-time/short-term parameter characterizations are comprised of descriptions that capture quick changes, such as changes in interval length, slope, and amplitude. Fast timescale information can indicate when, temporally and/or spatially, unusual changes in one’s internal state occurs. Such unusual changes in one’s internal state can be externally-event driven or internally-thought driven.

For daily-life monitoring applications it is of interest to characterize both long-term and short-term measures to identify emotional state and temporal/spatial events of interest, respectively. In light of these real-life monitoring concerns, four long-term and short-term IBI, blinking, brain activity, and skin conductance parameter characterizations are compared. Long-term parameter characterizations for IBI, blinking, brain activity, and GSR consist of the low frequency high frequency (LFHF) time-frequency ratio, count of blinks, frontal EEG alpha power, and tonic skin conductance level (SCL). Similarly, short-term parameter characterizations consist of the inverse of IBI (median RRI), significant and fast slope related changes in blink rate, asymmetric frontal EEG alpha power, and significant and fast slope related changes in GSR. In addition, understanding the relationship between long-term and short-term measures can help to distinguish between ambiguous behavior.

### Emotional state monitoring during navigational tasks

Navigation is the process of planning a path from a start to a goal location and driving through it with adequate velocities and accelerations keeping distance from obstacles. In terms of passenger transport, both automobile and PMV, the control and quality of navigation facilitates the user’s experience. There is increasingly more studies on passenger experience/feeling during automobile driving and riding, because even though the rider is just sitting (“doing nothing”) the passenger’s safety depends on the performance/direction of the vehicle. In car focused literature, the shift of the role of the human from a driver to a passenger is described as the loss of controllability. With the loss of control, there is a need for understanding/quantifying the passenger’s comfort and improving vehicle movement (considering safety and the rider’s expectations) [32]. More specifically, riders physiologically experience greater arousal than drivers, due to the possibility of loss of control [9]. Therefore, there may be a possibility for a similar phenomenon (a loss “sense of controllability”) during PMV riding. It is of interest to investigate a PMV user’s sense of control, as there is little literature on the topic.

In addition, it would be useful to quantify a PMV user’s physiological response in order to better assist with comfort and improved life quality. There are previous works, utilizing PMVs, in comfort factors during navigation have used self driving data to elaborate self-driving models for personal mobility vehicles where the system was evaluated based in passenger subjective measurements [6, 33]. In a vehicle navigational context, one approach for navigation evaluation based on passenger subjective and physiological measurements was presented in [7]. Different from navigation in a human-robot interaction context, a robotic manipulator handing-over task evaluation was based on physiological and subjective measures [34].

In this work, two different modalities for emotional state monitoring were evaluated: presence and absence of environmental stressors. The presence of a sequence of environmental stressors was investigated during autonomous riding and self driving, for passive riding and active driving response measurements. In autonomous riding the vehicle is programmed to perform all driving functions from a start to a goal location. By design, safe operation rests solely on the automated vehicle system. To achieve this, the vehicle localizes itself towards a previously built environmental map, plans a path from start to goal and computes appropriate velocities and accelerations to execute the path. In self driving the human knows the route to traverse beforehand, and operates the powered wheelchair joystick. The human is in complete control of the wheelchair’s navigation at all times, and is solely responsible for monitoring its safe operation.

Similarly, the absence of environmental stressors was tested using feedback relaxation/resting; homeostasis response measurements were considered. During feedback relaxation, before trials, we encourage participants to return to a standardized baseline in order to; compare emotional responses due to stressors between participants, gather steady-state arousal-valence information, and ensure that habituation effects were only due to trial exposure.

In addition, we organized data analysis of physiological measures in terms of capturing changes in emotional state over short/quick and long/slower time-scales.

1. Short-term parameter characterizations were composed of descriptions that capture quick changes, like changes in measurement interval length, slope, and amplitude.
2. Long-term parameter characterizations were composed using the mean or group methods and frequency domain analysis, thus capturing slower changes.

Such organization of parameter characterizations has not been demonstrated in previous work. In this work we identify which signal measures and characterizations are most descriptive for emotional state prediction in response to stressors, using both correlation and supervised classification, to assist with future modeling.

## Methods

### Participants

Fifteen healthy external Japanese participants (given monetary reward) with normal corrected vision [mean age, 22 ± 2 standard deviation (SD) years; males = 8, females = 7] performed the wheelchair task. All participants were right-handed, had little experience with wheelchair operation, and did not have endocrine or stress-related abnormalities. The Advanced Telecommunication Research Institute International Ethics Committee for the Department of Dynamic Brain Imaging approved the wheelchair riding/driving experiment and all participants gave written informed consent before the experiment. The Advanced Telecommunication Research Institute International Ethics Committee for the Department of Dynamic Brain Imaging is in alignment with the Declaration of Helsinki.

### General procedures

#### Experimental design

In the study two modalities were tested to quantify changes in emotional state during the presence and absence of environmental stressors: passive-active movement tasks in an indoor driving loop and feedback relaxation. The effects of habituation were investigated for active driving; habituation is a decrement in behavioral response due to repeated exposure to a stimulus [21, 35, 36]. Whereas, the “sense of controllability” was investigated for passive riding.

Passive riding was administered prior to active driving, to ensure that the effects of habituation were solely due to active driving and not order effects. In this regard, the comparison of passive riding to active driving is biased. Despite the fixed nature of the design paradigm, we briefly report on arousal effects due to autonomous riding and give suggestions for future non-confounded experimental design. The experiment compared the effects of the two modalities on HR, brain activity, GSR, blinking, and subjective reports of external stressors.

Autonomous riding consisted of participants passively sitting on a powered wheelchair that autonomously drove on a predefined path at a pre-planned speed. Self-driving consisted of participants actively controlling an electric wheelchair with a joystick. There were two types of corridor conditions during the driving modality; the narrow and wide corridor width was ∼1.4*m* and ∼2.0*m* respectively.

For self-driving, we hypothesized that participants would become habituated/accustomed to external stimuli, across loops, and this effect would be seen in both their driving performance and physiological measures. In addition, narrow corridors should give rise to increased arousal as opposed to wide corridors during active driving.

In between each movement task participants were encouraged to return to a relaxed mental state, which served as a baseline, via feedback from a meditation score device (Neurosky Mindwave) which is a single contact EEG sensor, where attention and meditation behavior is estimated via data classification methods [37]. Neurosky’s meditation score was used as a trusted reference for relaxation as reports have verified its reliability [38]. Relaxation is an emotional state having positive valence and low arousal. Returning participants’ mental state to the same baseline ensured consistent changes in behavioral response across task, despite the unconstrained nature of the experiment.

#### Experimental procedure

**Fig 3:**
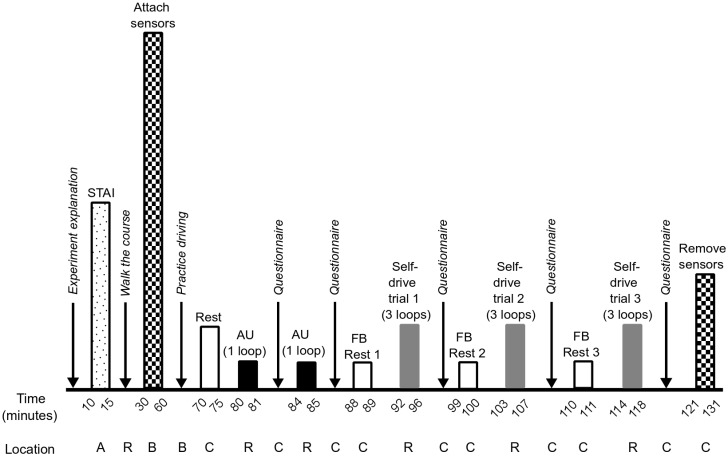
Experimental task paradigm. A time-line of task events, event timing, and event location are shown from left to right.

The experiment required participants to stay for three hours; three experiments were conducted everyday for one-week. Fig 3 displays a time-line view of the experiment. Upon arrival, participants were escorted to Room **A** where they gave informed consent and completed the Japanese version of the State-Trait Anxiety Inventory (STAI) self-evaluation questionnaire [39]. The STAI score was used to determine participant’s present anxiety level prior to the task.

**Fig 4:**
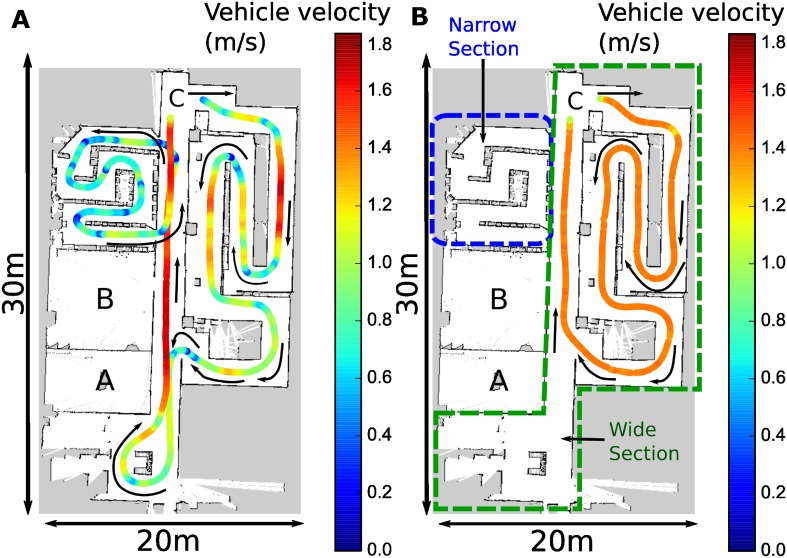
Top view of wheelchair experimental driving task path with its corresponding velocities for a typical participant run. The left figure, denoted by (a), depicts the self driving wheelchair path (length of approximately 150 m). The right figure, denoted by (b), shows the autonomous wheelchair driving path (length of approximately 80 m); wide and narrow sections are denoted by the green and blue dashed areas respectively.

Following, participants were informed about the passive and active riding tasks. First, the experimenter gave each participant an explanation of the self-driving course as they walked-through it (shown in Fig 4(a) and referred to as **R** for Run). After the walk-through, participants were escorted to Room **B** and asked to sit on the self-driving wheelchair where four non-invasive electrophysiological measurement devices were attached to the participant; a HR sensor, the eSense skin conductance sensor, a 6-channel EEG unit and the Neurosky Mindwave EEG device. No instruction was given for how participant’s should move their eyes (free blinking was permitted); participants were asked to remain as motionless as possible during the experiment.

Each participant was instructed on how to manipulate the manual wheelchair joystick and asked to practice driving in circles in Room **B** for some minutes in order to get used to its the wheelchair control. Subsequently they were asked to sit on the autonomous powered wheelchair and were driven to the start location **C**, as shown in Fig 4(b). To ensure that participants started the first run in a restful state, a five minute resting period was administered such that participants fixated on a black cross on a gray computer screen.

In autonomous riding, participants were asked to sit on the wheelchair while it navigated autonomously two times replaying a trajectory recorded by an expert who drove at constant velocity and with few jerks. Participants were given instructions on how to use a lever fixed to the wheelchair in order to report periods of perceived external stressors and the corresponding intensity. In the first autonomous run, participants were instructed to move a fixed lever on the wheelchair if they experienced negative valence such as fear or anxiety. During the second autonomous run, instructions were such that the lever could be moved if both positive and negative valence was experienced. To ensure participant safety, an experimenter equipped with an emergency stop button walked behind all riding participants. A run during autonomous riding is shown in Fig 4(b).

After the second autonomous run, participants were instructed to sit on the self-driving wheelchair for the active driving experiments. Each participant drove the wheelchair manually for three trials composed of three course loops each (a total of nine loops) starting from location **C**. They drove at their own comfortable driving style and velocities. Before each of the active trials a one-minute resting interval was administered, such that participants sat comfortably on the wheelchair and fixated on a black cross located in the center of a gray computer screen. The color varied from white to black, where white and black corresponded to relaxation and non-relaxation respectively via the Neurosky Mindwave EEG meditation score [37]. Participants were encouraged to use the cross color feedback, to ensure that their physiological response returned to a relaxed baseline. In addition, participants were encouraged to relax via auditory feedback. Auditory stimulation for 2 secs (seconds) with a tone of 2000 Hertz (Hz) frequency and 80 decibel intensity, at intervals of at least 16 secs was reported to enhance global alpha activity [40].

After each of the two autonomous loops and each of the three self driving trials, a nine question questionnaire was administered to determine the participants’ level of comfort. Each question could be rated from 1 to 7, where 1 and 7 corresponded to uncomfortable and comfortable. The questions consisted of the following topics: wheelchair comfortableness, wheelchair smoothness, wheelchair reliability, wheelchair speed, wheelchair motion, wheelchair navigation feeling, predictability of the motion, enjoyableness, practicability. At the end of the experiment, participants were asked to share their opinions about the experiment. Finally, sensors were removed and participants returned to Room **A** for monetary reward.

### Experimental equipment

**Fig 5:**
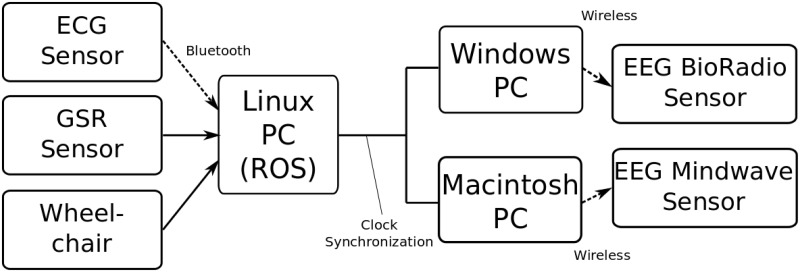
Data collection hardware connection diagram.

**Fig 6:**
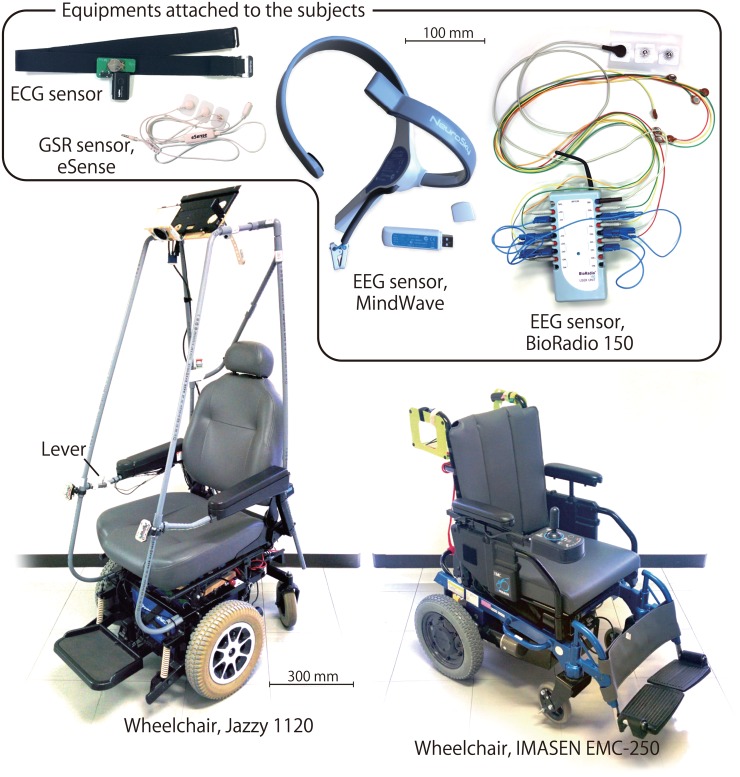
Equipment used during experimentation. Jazzy wheelchair (left) for the autonomous riding, IMASEN wheelchair (right) for the self-driving, and sensors attached to the subjects for the both tasks.

Four commercial devices were used to capture changes in physiological measures. Fig 5 shows a block diagram of the hardware connectivity during experimentation and Fig 6 shows an illustration of the sensing devices.

The BioRadio 150 Cleveland Medical Inc. wireless data acquisition system was used to record EEG from 6-location; F3, FC3, C3, F4, FC4, C4, Fpz (GND). Nihonkohden cupped electrodes with electrode paste was used to ensure reliable collection of EEG activity. The accompanying Windows based software, BioCapture, was used to record and save participant data via a Windows laptop at a sampling rate of 256 Hz. The dry single contact EEG based NeuroSky Mindwave system was used to capture blinking information and measures for general behavior, such as relaxation and attention. The Mindwave wirelessly connected to a Macintosh laptop, and a Java-based program was used to stream the data to a text file at 128 Hz using JSON format.

A Bluetooth ECG sensor using a soft strap by Polar Electro was used to measure inter-beat (RR) interval and its variability (see Fig 6). The sensor measures ECG signal around V1–V2 at a frequency of 48 kHz through bluetooth A2DP protocol.

Skin conductance was measured using the eSense by Mindfield/Germany, the sensor applies a small electric voltage to the skin and measures changes in resistance on the skin’s surface. The electrodes were applied to the tips of the index and middle fingers of the left hand. The eSense was connected to the microphone port, of a Linux laptop.

The eSense, HR sensor, and wheelchair measures were integrated using ROS (Robot Operating System) to ensure proper data alignment during data collection; the sampling rate was 10 Hz. Both the BioRadio and Neurosky were not integrated into ROS, thus both computer clocks were synchronized to the Ubuntu computer running ROS to ensure data could be aligned using timestamp information.

Two powered wheelchairs, the IMASEN EMC-250 and Jazzy 1120 by Pride Performance Mobility, were used in self-driving and autonomous riding tasks respectively. The IMASEN wheelchair has two powered rear wheels and two free casters in front, its maximum traveling speed is 1.6 *m/sec*. Similarly, the Jazzy wheelchair has two powered rear wheels and can reach speeds up to 4.0 *m/sec*. The Jazzy wheelchair is controlled based on ROS framework during the autonomous modality. To have consistent and reproducible runs, the authors used a motor controller TF-2MD3-R6 by T-frog Project that has dynamics compensation and an open-source motion controller ROS node “trajectory tracker” developed by the authors. This module controls the robot’s angular velocity such that a preliminary pre-recorded path trajectory was played back based on [41]. The trajectory tracker source code was implemented using ROS framework and is available at: (https://openspur.org/~atsushi.w/packages/).

### Physiological measures

In this section, we describe in detail the construction of both long-term and short-term characterizations for all four measurements (GSR, IBI, blinking, EEG).

#### Long-term characterizations

We characterized long-term blinking and GSR measurements using grouping methods, such as counting, and averaging. Long-term IBI and EEG measures are characterized using frequency power spectral density (PSD) of a relevant interval of frequencies.

- Blinkcount: Blinking was characterized as the count of blink events at intervals of 2 secs.
- GSRSCL: Skin conductance was characterized as the tonic/baseline level of electrodermal activity.
- LFHF: Changes in IBI are periodic and occur on many time scales. Quantification of these fluctuations can be achieved by computing the PSD for four frequency bands; ultra-low frequency (ULF) from 0-0.0033 Hz, very-low frequency (VLF) from 0.003-0.04 Hz, low frequency (LF) from 0.04-0.15 Hz, high frequency (HF) from 0.15-0.4 Hz. ULF and VLF require long term data for accurate characterization, therefore we focus on the ratio LFHF which represents sympathetic and parasympathetic equilibrium. We calculated LFHF using the following steps: window the IBI signal every 20 secs, calculate PSD for each windowed signal for LF and HF desired bands, apply a weighted windowing function (Hamming) to reduce spectral leakage, utilized the Welch method, computed the ratio. The Welch method separates the data into overlapping segments, in order to reduce the variance of the periodogram [22].
- Meditation: EEG frontal alpha power (8-13 Hz) and the proprietary meditation score was directly obtained from the Neurosky Mindwave. As previously mentioned, Meditation score is used as a trusted reference for general relaxation because previous reports verified its reliability [38, 42].

#### Short-term characterizations

Short-term blinking rate and GSR measures were characterized by determining significant slope increases [24]. IBI was found to be the most effective way to capture signal fluctuations. And, hemispheric differences in EEG alpha power was utilized to short-term behavior.

- GSRsiginc: GSR measures were smoothed by averaging neighboring points (running mean). Temporal locations where the smoothed GSR signal slope significantly increased were found. A square-wave signal was constructed such that each square-wave location corresponded to the temporal location of significant slope increase; otherwise a value of zero was assigned to the square-wave signal. The height and width of each square-wave corresponded to the maximum amplitude of the GSR signal during slope increase and the duration in which significant slope increase occurred, respectively.
- Blinksiginc: Blink rate was calculated by smoothing (averaging neighboring points) the Mindwave blink strength measure for an interval 0.1 secs; Mindwave blink strength is recorded as a point from 0 to 255 where a weak and strong blinks refer to values near 0 and 255 respectively. Upon detection of significant slope increase, the amplitude and interval width were used to create a square-wave signal capturing the location and amount of instantaneous signal change.
- medianRRI: IBI is characterized by the time difference between consecutive R peaks (RR interval) from the electrocardiogram signal [22]. The inverse of IBI, called median RRI, was used to standardize parameter meanings such that higher values imply more arousal and lower values imply less arousal.
- alpha-asymmetry: EEG frontal asymmetric alpha power was calculated by subtracting the alpha power recorded at the left hemisphere from the alpha power recorded at the right hemisphere [18].

## Analysis

This section presents three mathematical methods used to quantify physiological measures during the experimental paradigm. Correlation maps between physiological measurements were built to show the correlation between measurements in different time periods (short and long term). Time-frequency analysis was performed on the six-channel BioRadio EEG data in order to confirm the presence of emotional state related trends due to stressors. Unsupervised classification analysis was utilized to predict emotional states when correlation proved uninformative. In addition, one-way ANalysis Of VAriance (ANOVA) was used to describe changes in characterized parameters across feedback relaxation sessions and differences in parameters between experimental conditions. Significant effects (p-value < 0.05) were further investigated using Turkey’s honest significant difference criterion (HSD) post-hoc test. Self-report questionnaire distribution values were gaussian corrected, and significance was evaluated via sign tests and Wilcoxon signed rank test.

### Correlation map

For all tasks (self-driving, feedback relaxation, and autonomous riding) correlation analysis between parameter characterizations was performed. Each participant’s signal measures were tested for normality using the single sample Kolmogorov-Smirnov goodness-of-fit hypothesis test. Parameters with skewed distributions were transformed using the one-parameter Box-Cox transformation, such that $yt_{i}=\frac{y_{i}^{\lambda }-1}{\lambda }$ for *λ* ≠ 0. For *λ* = 0, the natural log of the data was used where *yt*_*i*_ = *log*(*y*_*i*_) [43]. Afterwards, Spearman correlation was calculated for both long-term and short-term characterizations, for each task. Significant correlations were determined by the corresponding p-value < 0.001 and correlations greater than 0.5 that were directly/primarily or secondarily associated with the evaluation lever. Delays from either data acquisition or physiological effects were considered by using computing the autocorrelation for varying time-shifts.

### Time-frequency

For all tasks (self-driving, feedback relaxation, and autonomous riding) time-frequency analysis of EEG data was performed, however self-driving is only reported in detail. Time-frequency analysis was performed on the EEG data for three frequency bands (low alpha, high alpha, beta), to determine whether one frequency band and or channel location was more useful in terms of emotional state prediction than another. Similarly, changes in channel location and frequency band were considered across trials. EEG data was first bandpass filtered between 8 and 25 Hz, and thresholding was done to remove artifacts. Time-frequency analysis was used to create a matrix of frequencies across time at intervals of 0.1 Hz. Frequencies were condensed into the previously stated three bands of interest.

Signal slope and variance measures were calculated for each band, channel, and condition (wide and narrow self-driving paths) in order to reduce the frequency information in terms of a scalar value. Slope was useful for capturing changes in each frequency band across trials. Variance was useful for capturing the magnitude of each band given that we were only interested in capturing the presence of large spectral amplitudes. All channel variance measures’, for each band and condition, were compared using the *z*-score to determine which variances were significantly large, denoting significant channel activation. The absolute value of the *z*-score was calculated for each using the standardized *z*-score, such that $\left|z\right|=|\frac{r_{k}-\bar{r_{k}}}{std(r_{k})}|\in R^{k\times 1}$ where *k* = 1, 2, …, *N* and *N* is the total number of datasets. $r\in R^{k\times 1}$ represents a general vector of length *k*, the overbar represents the mean, and *std*(·) represents the standard deviation. Significant variances (|*z*| < 2) were assigned a value of one, thus important channel activation could be counted for each frequency band and condition.

### Classification

For self-driving only, classification analysis was used to predict emotional state because correlation analysis was hypothesized to be weaker/ambiguous for prediction of a stressor. The hypothesis that correlations between measures would be weaker was based on previous reports where vehicle drivers showed less GSR, muscle tension, and HR activity than vehicle passengers [8, 9]. We inferred that measurements with reduced signal response would need more predictive modeling methods and additional measures such as EEG may assist with identifying changes in emotional state in response to stressors.

We aimed at determining stressor intervals by classifying the EEG data during those periods into presence of a stressor versus absence of a stressor classes. Continuous six-channel BioRadio EEG data was first divided into segments for each self-driving loop (Fig 4(a)). For each channel, each loop segment was then divided into shorter equal-sized periods, where each period was referred to as a stressor-interval. EEG data was band-pass filtered between the range of 3-50 Hz, detrended, and baseline corrected for a period of 0-1 secs. Various stressor-interval durations (2, 4, 5 secs) were compared, in order to determine the optimal interval duration for each label. Each of these labels were independently used for classification. Ratio of power in alpha band (8-13 Hz) to power in frequency range 4-30 Hz for (a) each EEG channel (b) asymmetry information, which is obtained by subtracting channels on right hemisphere from corresponding channels on left hemisphere were used as features. Ten-fold cross-validations were performed using Linear Discriminant Analysis (LDA), Support Vector Machines (SVM) and Simple Logistic Regression (SLR) methods for all the cases [44].

Labels were constructed using short-term (GSRsiginc, medianRRI), long-term (GSRSCL, Meditation), and behavioral (Joystick manipulation) characterizations. All characterizations were pre-processed before computing the following label construction steps: a cutoff value was selected for assigning presence of a stressor and absence of a stressor, the stressor-interval length was set to 2, 4, or 5 secs, divided the preprocessed characterizations into equal-sized successive intervals where each interval was equivalent to stressor-interval length, the mean was calculated for each equal-sized successive interval, the mean of each successive interval was evaluated such that mean values greater and less than the cutoff value was assigned (absence of a stressor = 2) and (presence of a stressor = 1) respectively. GSRsiginc, medianRRI, GSRSCL, Meditation, and Joystick used the following cutoff values respectively; mean of the preprocessed GSRsiginc (≈ 0.3), median of the preprocessed IBI, mean of the preprocessed GSRSCL (≈ 0.5), 50, mean of the preprocessed Joystick *R*^2^ value. Similarly GSRsiginc, medianRRI, GSRSCL, Meditation, and Joystick used the following interval durations in secs respectively; 2 and 4, 2 and 5, 2 and 5, 2 and 5, 5.

GSRSCL, medianRRI, Meditation, and GSRsiginc characterizations were preprocessed such that characterizations were normalized from 0 to 1 for each participant. Joystick manipulation was preprocessed such that the y-position, forward and backward movement, was smoothed and the variability between the actual and smoothed y-position was computed. The hypothesis was that jerky hand movements correspond to moments in which stressors are present; smooth movements represent joystick manipulation in the absence of a stressor. In particular, smoothing was computed using a running mean over 2*M* + 1, where *M* = 5, successive points, such that *M* points were on each side of the current point. The variability between the actual and smoothed manipulations were calculated via the coefficient of determination, such that $R^{2}=1-\frac{\sum_{i}{\left(x_{i}-{\hat{x}}_{i}\right)}^{2}}{\sum_{i}{\left(x_{i}-\bar{x}\right)}^{2}}\in \left[0,1\right]$ where *i*, *x*, $\hat{x}$, and $\bar{x}$ represents the vector length, actual joystick movement, smoothed joystick movement, and mean of actual joystick movement respectively [45].

## Results

This section presents findings for the passive-active movement tasks and feedback relaxation. The following three subsections report results for the participant population average.

### Active movement task (self-driving)

During self-driving trials (1, 2, 3), long-term and short-term parameter characterizations did not strongly correlate with each other. The lack of emotional response correlation could be due to participants having full control of their speed and position. Therefore, other measurements (EEG) and analysis methods (supervised classification) were investigated to predict moment-to-moment emotional state in response to external stressors during self-driving.

#### Time-frequency

Time-frequency analysis across trials and loops reveal the occurrence of two brain patterns; greater variance was found in right channels at low alpha frequencies as opposed to corresponding left channels at similar frequencies (denoted with underline in Table 1), and greater variance was found in left channels at higher alpha and beta frequencies as opposed to corresponding right channels at similar frequencies (denoted with parenthesis in Table 1). Table 1 shows the significantly counted frequencies per channel for both wide and narrow loops. In particular, during narrow corridor driving as opposed to wide corridor driving these findings are stronger in the sense that all hemisphere channels exhibit the same pattern instead of only particular channels. The right hemisphere alpha pattern is in alignment with stress-related EEG asymmetric reports, such that greater left alpha power is associated with avoidance [18, 30]. Greater emotional response due to perceived stressors are noted for narrow/constrained path driving than wide/open path driving, confirming previous reports that corridor related aspects can influence internal state response [46]. In addition, the left hemisphere high alpha and beta pattern is in alignment with situational anxiety EEG related reports [47]. Situational anxiety is a state of apprehension, discomfort, and anxiety caused by a new or changing event. These findings suggest that EEG measurements captured internal state response due to perceived stressors, and would thus be a reliable measurement to use in classification analysis for prediction of the presence of a stressor.

**Table 1:** Counted frequencies per channel based on variance for wide and narrow loops.

		*Electrode channels*					
*Condition*	*Freq. band*^a^	*F3*	*FC3*	*C3*	*F4*	*FC4*	*C4*
*Wide*	*α*_1_	**4**	4	2	2	5	4
	*α*_2_	1	4	3	(**4**)	0	6
	*β*	3	2	2	(**5**)	1	1
*Narrow*	*α*_1_	**5**	**9**	**6**	3	5	2
	*α*_2_	1	0	2	(**2**)	(**2**)	(**5**)
	*β*	1	1	3	(**3**)	(**1**)	(**4**)

*^a^α*_1_, *α*_2_, and *β* denote the following frequency ranges 8-12 Hz, 13-15 Hz, and 16-20 Hz, respectively.

#### Supervised classification

The goal of this analysis was to build a supervised classification two-class (presence of a stressor and absence of a stressor) model using EEG data during realistic self-driving exposure conditions. However, this is problematic because during realistic (non-stimulus induced) conditions it is unclear as to when and how long each participant perceives a stressor. In order to remedy this problem we constructed several labels (1 = presence of a stressor, 2 = absence of a stressor) that identified intervals of emotional arousal and valence change for each participant, trial, and loop. We tested two short-term characterizations (GSRsiginc, medianRRI), two long-term characterizations (GSRSCL, Meditation), and one behavioral (Joystick manipulation) labels. Table 2 shows LDA classification accuracy values (mean, standard deviation) for each label per trial, using all EEG channel and asymmetry information (L-R).

**Table 2:** LDA classification results during wide path self-driving.

		*Trial 1*		*Trial 2*		*Trial 3*	
*Label*^a^^b^		*M*	*SD*	*M*	*SD*	*M*	*SD*
GSRsiginc*(4)	*All ch*.	**69.7**	**11.9**	64.5	17.5	58.3	15.2
	*L-R*	53.9	2.7	50.1	11.5	49.8	11.5
GSRsiginc*(2)	*All ch*.	**63.9**	**7.5**	65.4	10.2	59.4	8.7
	*L-R*	52.4	14.3	52.9	10.8	52.5	12.9
GSRSCL^†^(2)	*All ch*.	58.9	13.2	54.7	13.2	52.4	10.8
	*L-R*	52.4	14.3	52.9	10.8	52.5	12.9
Meditation^†^(5)	*All ch*.	53.2	22.1	66.6	16.3	**67.2**	18.1
	*L-R*	58.4	4.8	65.7	4.5	59.9	4.6
Joystick^‡^(5)	*All ch*.	54.8	13.7	54.0	13.9	**60.6**	13.2
	*L-R*	53.3	17.9	51.3	21.9	54.7	17.1
medianRRI*(2)	*All ch*.	57.9	11.3	54.9	14.1	56.4	11.8
	*L-R*	53.9	2.7	50.1	11.5	49.8	11.5

^a^*, †, and ‡ denote short-term, long-term, and behavior related labels, respectively.

*^b^*The parenthesized number following the label name denotes the interval duration in secs.

EEG data during wide path driving was only considered for classification analysis, as opposed to narrow path driving data, because EEG data during narrow path driving was too short and training/test sets could not be properly constructed due to lack of data. Treating narrow and wide paths separately also improved the consistency of performance as compared to when both were combined. Data suggests that slower motion evokes different physiological responses over different lengths of time in comparison to faster motion. The prediction of emotional state due to a stressor at different speeds might benefit from using additional features that capture various physiological response trends.

Three classification methods (LDA, SVM, SLR) were used to identify the most reliable, in terms of above chance-level accuracy and reproducibility of accurate prediction, modeling method for stressor presence prediction. The three methods gave statistically similar accuracy results, however LDA often achieved higher accuracy, regardless of label type and stressor-interval duration, than the other two methods. A possible reason why LDA performed similarly to the other methods could be due to the fact that the data was generated from two multivariate Gaussian distributions with similar covariance matrices. In addition to identifying a reliable modeling method, each of the five labels were tested and compared with multiple intervals (2, 4, 5 secs) to determine the most optimal interval for prediction of a stressor per label. Results showed that labels generated with physiological measures (GSRsiginc, GSRSCL, medianRRI) achieved more reliable results with shorter (2 sec) stressor-interval durations, as opposed to longer intervals. However, labels based on non-physiological measurements like Joystick and Meditation produced notable results for longer (5 sec) stressor-interval durations. Thus, classification performance largely depends on the modality used to create labels as well as on the chosen duration of the stressor-interval.

Overall, in terms of the most reliable label for accurate stressor presence prediction, the short-term GSRsiginc label achieved the highest accuracy results in comparison to the other labels. GSRsiginc (2 secs) predicted similarly to GSRsiginc (4 secs), however the 2 sec stressor-interval predicted more consistently amongst participants. To emphasize this point, both means and standard deviations for GSRsiginc (2 secs) and GSRsiginc (4 secs) are shown in bold font in Table 2 for trial 1 only. In addition, the utilization of all EEG channels, as opposed to channel asymmetry, resulted in higher prediction accuracy for all labels. We suspect that the difference in accuracy is due to the fact that the usage of all-channels incorporates the main trend of asymmetric differences. This observation is in alignment with the time-frequency analysis, thus further confirming that significant asymmetric brain activity in alpha range existed.

For all of the labels, except for Joystick and Meditation, the prediction accuracy decreased across trials. IBI and GSR measurement amplitudes decreased across trials due to habituation effect, therefore we suspect that the label may be becoming less effective due to the decline in behavioral response. Meditation and Joystick accuracy might increase because they are not based on physiological measurements, that are prone to habituation. Additionally, the Meditation label prediction accuracy increased over trials, suggesting that participants are generally feeling more relaxed during driving and are thus becoming habituated/familiar to the situation. The accuracy for the Meditation label during trial 3 is shown in bold font in Table 2.

Finally, the usage of behavioral labels have the potential to test for other useful conditions, besides the presence of a stressor, such as participant skill-level, fatigue, and/or personality traits. The prediction accuracy for Joystick increased across trials; explicitly suggesting an improvement in driving skill and implicitly suggesting increased relaxation according to our assumption. In Table 2, we highlight the Joystick mean accuracy for trial 3 to emphasize this point.

### Passive movement task (autonomous riding)

The autonomous modality included three unique aspects; 1) few artifacts included in measurements because participant’s were not responsible for driving and could sit without moving, 2) participants used a lever to continuously report his/her current feeling of emotional state relative to their previous state (starting point was from an arousal and valence state of zero), 3) participant’s physiological responses exhibited a clear trend and changed dynamically. However, it is unclear whether physiological responses are purely due to a loss of controllability, ordering effects, or other causes.

**Fig 7:**
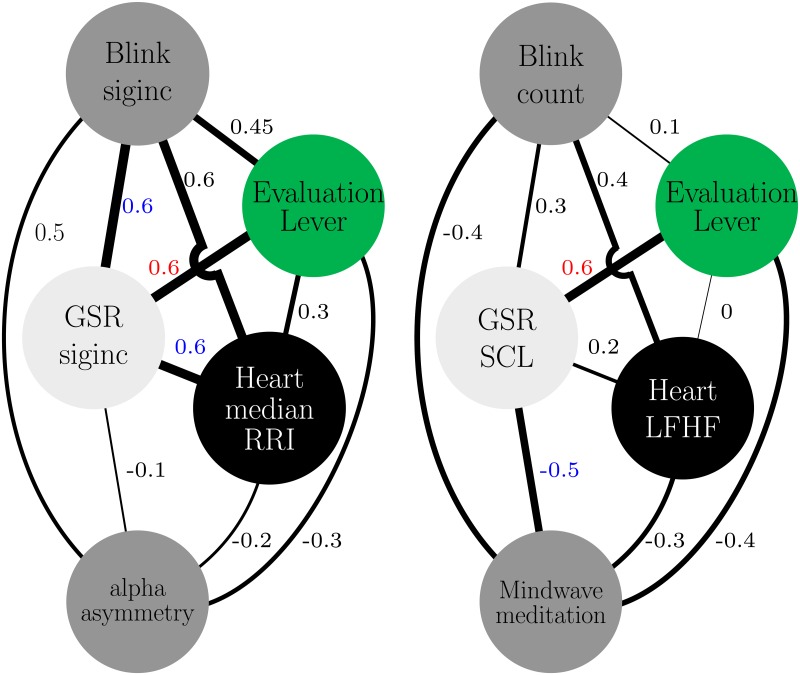
Short-term (left) and long-term (right) correlation maps during autonomous riding. Significant primary and secondary correlations with respect to lever evaluations are shown red and blue, respectively. The width of the connection line corresponds to the Spearman correlation *ρ* value, solid lines indicate significant correlations.

Short-term parameter characterizations more accurately captured periods of perceived stressors, as opposed to long-term parameter characterizations, according to participant evaluation lever reports. In particular, both short-term and long-term parameters, skin conductance characterizations correlated strongest with stressor lever reports, as shown in Fig 7. Significant Spearman correlations that were greater than 0.5 and were directly/primarily or secondarily associated with the evaluation lever were considered. However, despite the fact that subjects were asked to continuously rate their perceived change in emotional state from their previous state, as shown in Fig 7, we can not be certain of the subject’s response in relation to another situation. The subject’s responses of what is consided/perceived to be a stressor in this experiment may differ if the event/curcumstance was different. The stressors of the experiment in relation to a real-life stressor, may not be comparable because subjects were aware that the experiment was safe and simulated.

**Fig 8:**
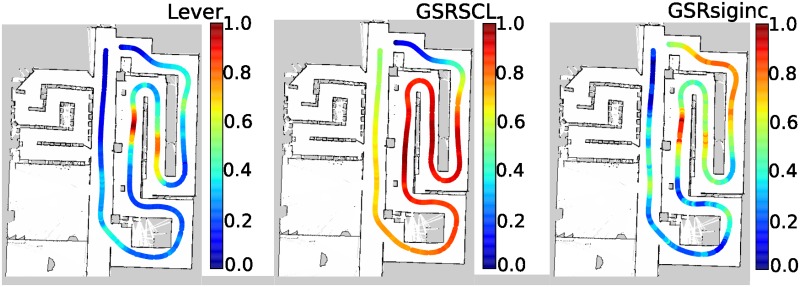
Evaluation lever and GSR characterizations during autonomous riding.

The short-term GSRsiginc characterization (*SD = 0.14*) closely resembled the evaluation lever reports (*SD = 0.06*), the normalized mean is shown in Fig 8. Whereas, the long-term GSRSCL characterization (*SD = 0.05*) could only accurately capture the initial stressor instead of multiple stressors, due to the slow change in basal level of the physiological signal. This demonstrates and confirms that skin conductance alone can be used to reliably detect the presence of stressor events, in particular the short-term characterization has the potential to detect stressor events in real-time during autonomous riding.

In order to improve the reliability of predicting the presence of a stressor, it is of interest to understand the relationship between other physiological measures. Short-term Blinksiginc, medianRRI, and GSRsiginc characterizations strongly correlated (*ρ* = 0.6; *p* < 0.001) with each other, suggesting that monitoring the relationship between these secondary parameters could potentially detect the presence of real-time stressors. It is plausible for such a relationship to exist because the characterizations capture incremental changes in nervous system related measures. The GSRsigninc characterization correlated strongest with the evaluation lever because the lever and GSR recording delay was similar, within 2 secs. Heart rate and blinking was 6.5 secs and 4.65 secs behind the evaluation lever respectively. In addition, long-term GSRSCL and Meditation had a strong negative secondary correlation, confirming the reliability of the meditation score [38]. During periods where stressors were present, GSRSCL increased and slowly decreased, and similarly Meditation decreased and slowly increased.

### Feedback relaxation (resting)

Before self-driving trials, we encouraged participants to align with the emotional state of relaxation by viewing their Mindwave score in terms of fixation cross color; white and black corresponded to relaxed and not relaxed (attentive). Parameter characterizations that significantly and strongly correlated with the Mindwave score were considered reliable characterizations for capturing emotional state, in particular relaxation. Meditation score was used as a trusted reference for general relaxation based on our previous experiences and previous reports verified its reliability [38, 42].

**Fig 9:**
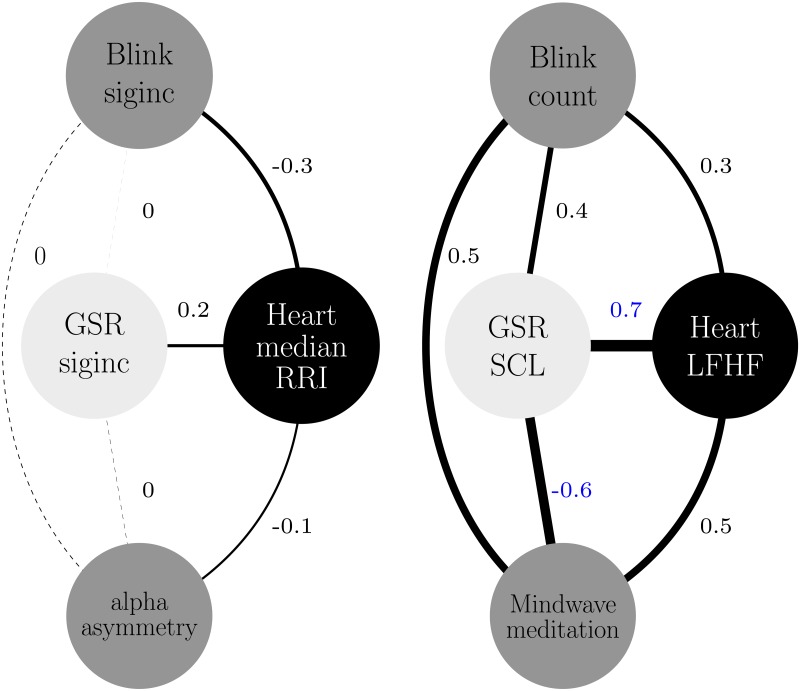
Short (left) and long (right) term correlation map during initial rest. Significant secondary correlations with respect to lever evaluations are shown in blue. The width of the connection line corresponds to the Spearman correlation *ρ* value, dashed and solid lines indicate significant and non-significant correlations respectively.

During feedback relaxation periods (sessions 1, 2, 3) long-term parameters GSRSCL and LFHF (*ρ* = 0.7, 0.8, 0.6; *p* < 0.001) and GSRSCL and Meditation (*ρ* = 0.6, 0.4, 0.8; *p* < 0.001) strongly and significantly correlated with each other. Conversely, short-term parameters weakly and/or non-significantly correlated with Meditation and with each other. Fig 9 shows the correlative relationship for the first session of feedback relaxation. This suggests that the long-term characterizations reliably convey information about slow changes in emotional state. In addition, the mean of long-term parameter characterization correlations slightly increased across sessions. Using one-way ANOVA and post-hoc test TurkeyHSD, session 1 (*p* < 0.16) and session 2 (*p* < 0.02) correlations were smaller/weaker than session 3 correlations. Suggesting that practiced repetition of emotional states could strengthen synchronicity of bio-signals, perhaps allowing for better prediction of behavior.

**Fig 10:**
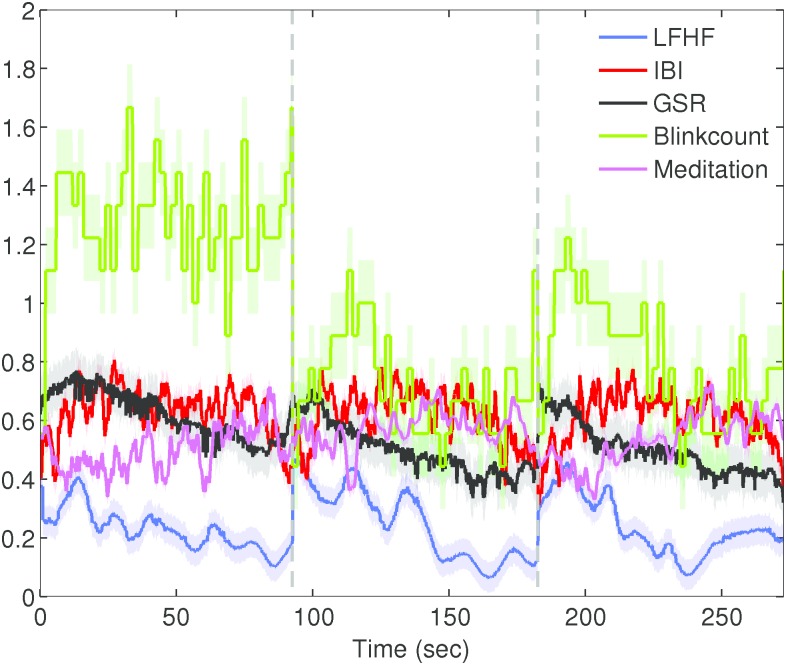
Long-term characterizations for each resting session.

IBI and Meditation increased across sessions; session 1 in comparison with session 3 reported significant increases (1-way ANOVA, TurkeyHSD; *p* < 0.009) for both parameters. Fig 10 shows each long-term characterization across rest sessions, each rest session is denoted by the dividing dashed-line. Conversely, GSRSCL and Blinkcount decreased across sessions; session 1 in comparison with session 3 revealed significance (1-way ANOVA, TurkeyHSD; *p* < 0.001) for both characterizations. This indicates that participants increasingly became more relaxed during resting sessions. Similarly, participants became more relaxed during resting sessions on average (1-way ANOVA, TurkeyHSD; *p* < 0.001). All three population resting sessions were averaged and divided in half, the first and last portions of resting were evaluated. GSRSCL and Blinkcount decreased, and Meditation increased significantly during sessions; suggesting attainment of a relaxed state. In addition, LFHF decreased and IBI decreased, implying that participants were inhaling often in order to assist with relaxation.

## Discussion

This work explored the utility and usage of several well-known physiological emotional state measures (GSR, HR, blinking, EEG) in the context of a realistic driving task, in order to assist with signal measurement and characterization selection before modeling of desired behavior/s. Physiological measures were divided in two categories: short-term and long-term to predict two-types of emotional state change: moment-to-moment and slower/gradual changes. The reliability of both short-term and long-term characterizations were validated using real-time subjective responses and a commercial software measure (Neurosky meditation score), respectively. We intended for this work to be an initial step for further investigation on the topic of emotional state due to stressors during wheelchair usage, as there is not much existing literature regarding passenger emotional response during wheelchair usage.

The idea of using multiple measures to improve the prediction of one’s internal state is not novel, however casting this concept into a framework in terms of physiological response scales (short-term, long-term) and physiological response delay is useful for application usage in various fields (robotics, engineering, etc). Such a framework can be useful for developers/researchers whom are not strictly focused on physiological characteristics and mechanisms, but are focused on research and development utilizing these physiological traits. For example, the development of wheelchair technologies that provide emotional state monitoring, of elderly and disabled individuals, could improve quality of life (subsection 0.6).

Application/product developers must consider the different cases of usage and design solutions (i.e.: predict user state) to address these cases. The results in this paper present information, via a developer focused framework, for different cases of wheelchair usage; active, passive, rest. During observed cases it is important to consider: type of measurement (non-physical versus physiological), characterization of measures (short-term versus long-term) (subsection 0.1), the relationship between measures (subsection 0.4), modeling of data based on speed, type of classification method, length of modeling interval. It is also important to consider habituation effects (subsection 0.2) and the users role in controlling the wheelchair (subsection 0.3). The usage of physiological measures for user state assessment is fairly novel, thus we compare its usage with (well-known, reliable) self-report methods (subsection 0.5).

Experimental design of a multi-condition experiment, while controlling habituation effects per condition, can be challenging. We choose to fix the design paradigm, such that autonomous was first and self-drive was second, because we were strongly interested in observing habituation effects across the loops and trials of the self-drive condition. We hoped to eliminate doubt that habituation during self-drive could be due to the ordering of conditions. On the down side, by fixing the condition order, we can not observe the effects of autonomous riding and self-driving independently. In order to remedy this problem, we suggest two options for future experimental design such that both conditions can be observed/compared independently while maintaining habituation effects: 1) two separate populations that only perform one of the conditions (self-drive only or autonomous only), 2) one population that performs one condition (self-drive only) on one day/week and then come back to perform the other condition (autonomous only) on another day/week. For the first suggestion, both populations would need to be statistically justified that they were similar.

### Multi time-scale characterization

Short-term characterizations (GSRsiginc, medianRRI, Blinksiginc) strongly and/or significantly correlated with real-time subjective reports of emotional state (Evaluation lever), as opposed to corresponding long-term characterizations. Thus validating that short-term signal characterizations reliably captured moment-to-moment changes of emotional state during autonomous riding. Similarly, short-term GSR, Heart, and Blink characterizations showed a strong correlation amongst each other as opposed to corresponding long-term characterizations. On the other hand, during self-driving short-term characterizations exhibited weaker correlations amongst each other, demonstrating that correlation alone cannot reliably predict the presense of a stressor during self-controlled driving/behavior. Long-term characterizations during feedback relaxation, especially GSRSCL and LFHF, strongly and significantly correlated with the Mindwave meditation score, demonstrating that these parameters best describe slow changes in emotional state due to stressors.

### Effects of habituation

We choose to use participants whom had “little experience” with “wheelchair operation” because we wanted to determine how quickly participants would adapt/habituate to the experimental stressors. Our goal was to quantify the changes in their emotional response as they became adapted/habituated to using the wheelchair. The purpose of looking at how people’s emotional response changed to stressors as they became habituated, was to determine which sensors and/or algorithms would be effective at predicting emotional state for experienced/habituated users (i.e.: disabled and/or elderly).

Habituation effects across self-drive loops and trials demonstrated that physiological measures become diminished over exposure and correlations in measurements decrease over long periods of time. In this case, short-term characterizations can be used in conjunction with classification analysis to predict moment-to-moment presence of stressors. We would expect similar reduction in physiological response to external stressors during passive riding. In addition, during periods of resting, habituation effects in response to repeated relaxation resulted in increased correlation/synchronicity of long-term measurement characterizations. Knowing trends in relations to habituation, can allow for better prediction of emotional state over long periods of time.

### Implications of controllability

It has been reported that humans express increased levels of stress when they are unable to control aspects of their environment. It is reported that humans who can not control threats produce the strongest stress responses, or increased emotional arousal and decreased valence state. Lack of control over the aspects of one’s environment may appear trivial, however it is psychologically important for health due to decreased stress, decreased disease, and increased life expectancy [14].

In the context of our experiment, participants are similarly unable to control the physical aspects of their environment (speed, direction, and acceleration of their movements), therefore it is plausible that the autonomous riding condition could cause more emotional response than the self-driving condition. During autonomous riding participant’s physiological responses exhibited a clear trend that correlated strongly with continuous self-reports of perceived stressors. This result is in alignment with previous reports where vehicle passengers showed increased skin conductance, muscle tension, heart rate response, and anxiety in comparison with drivers [8, 9]. However, it is uncertain whether physiological responses are purely due to a loss of controllability, ordering effects, or other causes. Therefore, additional experimentation should be conducted, such that autonomous riding and self-driving conditions are administered in randomized order such that loss of controllability can be more closely investigated. In addition, it is important to note that the stressors of the experiment in relation to real-life stressor’s may not be comparable because subjects were aware that the experiment was safe; the focus of the experimental paradigm was to understand trends within a predefined setting.

### Measurement and algorithm utility

We used supervised classification to assist in prediction of emotional state using EEG measurements. Time-frequency analysis of EEG measurements revealed that two emotional state related trends existed, thus EEG data could serve as reliable features to identify the presense of a stressor. The GSRsiginc label resulted in reliable classification of moment-to-moment emotional state, as opposed medianRRI, demonstrating that this procedure of model creation is plausible. We recommend the usage of realistic data, using additional feature sources, such as IBI, with EEG measurements in order to improve moment-to-moment prediction of the presense of a stressor with the GSRsiginc label. Common approaches on classification of emotional state due to stressors, identify periods of stressor presense and absense using stimulus induced conditions [25, 26].

In terms of measurement utility, skin conductance characterizations, both long-term and short-term, best captured changes in emotion due to stressors. This result is in alignment with related reports of reliable GSR characterizations to external stimuli [48]. For instance, short-term GSR correlated strongest with evaluation lever reports during autonomous riding, and long-term GSR correlated strongest with Meditation during rest and evaluation lever during autonomous riding. The second most useful measurement characterization was heart rate; short-term heart rate consistently correlated strongest with GSR during autonomous riding, long-term heart rate correlated strongly with GSR during rest. Blinking strongly correlated with GSR, however it was not as reliable because depending on each participant opposite trends were observed such as increased and decreased blinking during rest. EEG measures were the third most reliable measurement; trends were observable via statistical methods but weaker correlations with evaluation lever reports were obtained.

We anticipate that physiological measurements (GSR, heart rate) can be taken accurately while traversing rough terrains, however EEG and blinking would not be suitable due to increased signal artifact.

### Self-report in comparison to physiological measures

Eight of the fifteen participants showed significantly high state and/or trait anxiety before the experiment [39]. However, classification results for these participants were not different from participants that did not express state-trait anxiety. For self-reported questionnaires, participants reported the experience of faster wheelchair movement during autonomous riding as opposed to self-driving. Similarly, participants experienced a lack of being able to predict wheelchair movement during autonomous riding as opposed to self-driving. Both of these reports could be due to ordering effects because autonomous riding experiences were always before self-driving, where new situational experiences could give rise to increased emotional arousal. Nevertheless, these reports are in alignment with our findings that increased emotional state change was experienced during autonomous riding. However, participants also reported similar levels of comfort during autonomous riding and self-driving, which is in contradiction with physiological measurement findings. Qualitative statements, regarding whether participants had undergone a stressful experience, where not collected and participants are no longer available for further inquiry. We conclude that it is useful to collect physiological measurements, in addition to standard self-report questionnaires, to ensure that ambiguous findings are minimized. Physiological measurements were confirmed to be reliable because real-time continuous self-reports, during autonomous runs, showed emotional state change at similar locations and durations as short-term physiological characterizations. Therefore, it is of interest to use/develop physiological measurements for future emotional state prediction along with real-time continuous self-reporting methods, in addition to self-report questionnaires.

### Implications of utility for elderly and disabled individuals

Due to the immature/growing development of autonomous PMV technologies, there is little work regarding passenger’s emotional state while riding and/or self-driving such systems. Similarly, there is less related work using elderly/disabled users, because novel technologies are often tested on healthy-controls before being applied to the intended audience. Despite the fact that we used a younger test population, the knowledge regarding the sensor utility, algorithm utility, and sensor data time-scale framework can be re-used/condensed for other user populations. Final realization of this application for a specific population, would not be to use all physiological sensors for internal state monitoring. Rather, an ideal/feasible outcome would be the identification and usage of one or two physiological measures in combination with an effective algorithm, such that accurate predictive measurements for short-term and long-term emotional state could be quantified in response to external stressors.

With this goal in mind, we surmise that both autonomous and self-drive situations would result in lower correlation of physiological measures due to habituation (constant exposure) to external events from a fixed view-point. In addition, older individuals are less likely to be aroused by external stressors and are more positively valence biased, in comparison to younger individuals [49]. Therefore, accurate monitoring of comfort for long-term exposure (disabled and elderly users) would require user-specific models (via pattern classification and/or variable time-windowing of measurements) that are constantly updated, in order to reveal subtle changes in physiological measures. In addition, heart rate, temperature and/or skin conductance would be the most feasible types for non-invasive sensors for monitoring the nervous system. However, temperature and/or skin conductance sensors would require secure placement on the body, away from the hands, for realistic usage in daily-life. We demonstrated the usage of skin conductance measurement and characterization via finger-tip placement, however other types of sensor body placement (foot, forehead) are viable and need to be explored.

## Supporting Information

S1 File The S1_File.zip file contains a sample dataset of one subject. This file contains three separate files. Two excel files that display the wheelchair, GSR, heart rate (Dataset A in S1 File), and EEG (Dataset B in S1 File) data for each condition (Wide, Narrow), trial, and loop. The additional text file explains the organization of the data (File C in S1 File). The datashare file can be found at https://figshare.com/s/178c2b31710b69445d59. In case of any questions or inquiries please contact: yoichims@ieee.org.
(ZIP)
