# Supplementary material for: Multi-Sensor Based State Prediction for Personal Mobility Vehicles
Source: PLoS One. 2016 Oct 12;11(10):e0162593. doi: 10.1371/journal.pone.0162593 (PMC5061423; doi:10.1371/journal.pone.0162593)
Supplement: S1 File — This file contains three separate files. Two excel files that display the wheelchair, GSR, heart rate (Dataset A in S1 File), and EEG (Dataset B in S1 File) data for each condition (Wide, Narrow), trial, and loop. The additional text file explains the organization of the data (File C in S1 File). The datashare file can be found at https://figshare.com/s/178c2b31710b69445d59. In case of any questions or inquiries please contact: yoichims@ieee.org. (ZIP) [file pone.0162593.s001.zip › S1_File/FileC.rtf]

Order of the experimental events: 

(1a) num41_sub16_RE1 = rest

(1) num41_sub16_W_TR1_L1 = means subject number 16, performing self-drive for the Wide driving route, for TRial 1, Loop 1.  

(2) num41_sub16_N_TR1_L1 = means subject number 16, performing self-drive for the Narrow driving route, for TRial 1, Loop 1.

(3) num41_sub16_W_TR1_L2 = means subject number 16, performing self-drive for the Wide driving route, for TRial 1, Loop 2.  

(4) num41_sub16_N_TR1_L2 = means subject number 16, performing self-drive for the Narrow driving route, for TRial 1, Loop 2.

(5) num41_sub16_W_TR1_L3 = means subject number 16, performing self-drive for the Wide driving route, for TRial 1, Loop 3.  

(6) num41_sub16_N_TR1_L3 = means subject number 16, performing self-drive for the Narrow driving route, for TRial 1, Loop 3.

(7a) num41_sub16_RE2 = rest

(7) num41_sub16_W_TR2_L1 = means subject number 16, performing self-drive for the Wide driving route, for TRial 2, Loop 1.  

(8) num41_sub16_N_TR2_L1 = means subject number 16, performing self-drive for the Narrow driving route, for TRial 2, Loop 1. 

(9) num41_sub16_W_TR2_L2 = means subject number 16, performing self-drive for the Wide driving route, for TRial 2, Loop 2.  

(10) num41_sub16_N_TR2_L2 = means subject number 16, performing self-drive for the Narrow driving route, for TRial 2, Loop 2.

(11) num41_sub16_W_TR2_L3 = means subject number 16, performing self-drive for the Wide driving route, for TRial 2, Loop 3.  

(12) num41_sub16_N_TR2_L3 = means subject number 16, performing self-drive for the Narrow driving route, for TRial 2, Loop 3.

(13a) num41_sub16_RE3 = rest
(13) num41_sub16_W_TR3_L1 = means subject number 16, performing self-drive for the Wide driving route, for TRial 3, Loop 1.  

(14) num41_sub16_N_TR3_L1 = means subject number 16, performing self-drive for the Narrow driving route, for TRial 3, Loop 1.

(15) num41_sub16_W_TR3_L2 = means subject number 16, performing self-drive for the Wide driving route, for TRial 3, Loop 2.  

(16) num41_sub16_N_TR3_L2 = means subject number 16, performing self-drive for the Narrow driving route, for TRial 3, Loop 2. 

(17) num41_sub16_W_TR3_L3 = means subject number 16, performing self-drive for the Wide driving route, for TRial 3, Loop 3.  

(18) num41_sub16_N_TR3_L3 = means subject number 16, performing self-drive for the Narrow driving route, for TRial 3, Loop 3.


ODOM_GSR_HEART (Contained in the .xls file named ‘datashare.xls’ (yellow header), where each tab is for one condition (Narrow, Wide), one trial (1,2,3), one loop (1,2,3))

column 1		counter [counts the number of data samples]
column 2		unix_time: [Unix time(ms) in GMT/UTC timezone]
column 3		JST hour: [wall clock hours in JST timezone]
column 4		JST minute: [wall clock minutes in JST timezone]
column 5		JST second: [wall clock seconds in JST timezone]
column 6		JST millisecond: [wall clock milliseconds in JST timezone]
column 7		elapsed time (from 0 seconds) : [total appended time in seconds from start to end]
column 8		elapsed travel distance: [Travel distance(m)]
column 9		x position: [coordinate x(m)]
column 10		y position: [coordinate y(m)]
column 11		yaw: [yaw angle (rad)]
column 12		velocity: [velocity(m/s)]
column 13		angular velocity: [angular vel.(rad/s)]  absolute value
column 14		joystick x direction movement: [Joystick x]  -1<jx<1
column 15		joystick y direction movement: [Joystick y]  -1<jy<1
column 16		stop button push (0=did not push, 1=push)
column 17		Interbeat interval heart measure: [Heart R-R interval(sec.)]
column 18		Low frequency high frequency heart ratio: [LF/HF ratio]
column 19		GSR: [Skin conductance]
column 20		distance to objects: [Distance to obstacle in map(m)]
column 21		dir obj: [Direction of obstacle(rad)]

Note: * [x], [y], [yaw] are online estimated value. * [Distance to obstacle in map] is from the center of the wheelchair based on the estimated position. 0.35 to 0.4m of this value indicate the collision. * Analysis time window of [LF/HF ratio] is 25 seconds.


BLINK (Contained in the .xls file named ‘datashare.xls’ (blue header), where each tab is for one condition (Narrow, Wide), one trial (1,2,3), one loop (1,2,3))

column 1		counter
column 2		JST hour
column 3		JST minutes
column 4		JST seconds
column 5		JST milliseconds
column 6		blink strength (0 - 255)
column 7		mindwave attention score (0-100)
column 8		mindwave meditation score (0-100)
column 9		delta
column 10		theta
column 11		low_alpha
column 12		high_alpha
column 13		low_beta
column 14		high_beta
column 15		low_gamma
column 16		high_gamma


EEG (Contained in the .xls file named ‘datashare_EEG.xls’, where each tab is for one condition (Narrow, Wide), one trial (1,2,3), one loop (1,2,3))

column 1		JST time (string format)
column 2		F3
column 3		FC3
column 4		C3
column 5		F4
column 6		FC4
column 7		C4
